# Pancreatic cancer survival prediction via inflammatory serum markers

**DOI:** 10.1007/s00262-021-03137-6

**Published:** 2022-01-16

**Authors:** Mira Lanki, Hanna Seppänen, Harri Mustonen, Aino Salmiheimo, Ulf-Håkan Stenman, Marko Salmi, Sirpa Jalkanen, Caj Haglund

**Affiliations:** 1 Department of Surgery, University of Helsinki and Helsinki University Hospital, PO BOX 22, 00014 Helsinki, Finland; 2 Translational Cancer Medicine Research Program, Faculty of Medicine, University of Helsinki, Helsinki, Finland; 3 Department of Clinical Chemistry, University of Helsinki and Helsinki University Hospital, Helsinki, Finland; 4 MediCity Research Laboratory, University of Turku, Turku, Finland

**Keywords:** Cytokines, Inflammation, Pancreatic cancer, Pancreatic ductal adenocarcinoma, Prognosis

## Abstract

**Background:**

For prognostic evaluation of pancreatic ductal adenocarcinoma (PDAC), the only well-established serum marker is carbohydrate antigen CA19-9. To improve the accuracy of survival prediction, we tested the efficacy of inflammatory serum markers.

**Methods:**

A preoperative serum panel comprising 48 cytokines plus high-sensitivity CRP (hs-CRP) was analyzed in 173 stage I–III PDAC patients. Analysis of the effect of serum markers on survival utilized the Cox regression model, with the most promising cytokines chosen with the aid of the lasso method. We formed a reference model comprising age, gender, tumor stage, adjuvant chemotherapy status, and CA19-9 level. Our prognostic study model incorporated these data plus hs-CRP and the cytokines. We constructed time-dependent ROC curves and calculated an integrated time-averaged area under the curve (iAUC) for both models from 1 to 10 years after surgery.

**Results:**

Hs-CRP and the cytokines CTACK, MIF, IL-1β, IL-3, GRO-α, M-CSF, and SCF, were our choices for the prognostic study model, in which the iAUC was 0.837 (95% CI 0.796–0.902), compared to the reference model’s 0.759 (95% CI 0.691–0.836, NS). These models divided the patients into two groups based on the maximum value of Youden’s index at 7.5 years. In our study model, 60th percentile survival times were 4.5 (95% CI 3.7–NA) years (predicted high-survival group, *n* = 34) and 1.3 (95% CI 1.0–1.7) years (predicted low-survival group, *n* = 128), log rank *p* < 0.001. By the reference model, the 60th percentile survival times were 2.8 (95% CI 2.1–4.4) years (predicted high-survival group, *n* = 44) and 1.3 (95% CI 1.0–1.7) years (predicted low-survival group, *n* = 118), log rank *p* < 0.001.

**Conclusion:**

Hs-CRP and the seven cytokines added to the reference model including CA19-9 are potential prognostic factors for improved survival prediction for PDAC patients.

## Introduction

Pancreatic cancer is an aggressive disease and a worrisome cause of death. In 2018, the estimate was its causing nearly as many deaths (432,000) as cases (459,000) worldwide [1]. Among pancreatic cancer cases, pancreatic ductal adenocarcinoma (PDAC) accounts for over 90%. Curative treatment comprises radical surgery combined with oncological treatment, but only 10–20% of patients are surgically treatable at diagnosis [2, 3]. Even those who do undergo surgery have only a 25% chance of surviving for the next 5 years, with great variance in survival time [4].

The only serum marker for prognostic evaluation prior to surgery is carbohydrate antigen CA19-9, an established PDAC cancer marker. Like CA19-9, C-reactive protein (CRP) has also been associated negatively with survival [5, 6].

Inflammation and immunity display a complex relationship to cancer because they show properties that are both pro-tumor and anti-tumor [7]. Paramount in immune modulation are cytokines. The primary inflammatory cytokines TNF and IL-1 show pro-tumor effects, and IL-1 provokes metastasis [8]. The cytokines may originate from various cell types in the tumor and its microenvironment. Associated with tumor metastasis and recurrence, there has been a shift from T_H_1 to T_H_2 cytokines in the gene expression signature. [9]

Hence, the amount of detail that cytokines provide regarding inflammation may prove valuable. We investigated whether a detailed serum cytokine analysis can produce helpful information predicting survival, and whether we can find new potential prognostic factors to improve prediction.

## Materials and methods

### Patients

**Fig. 1:**
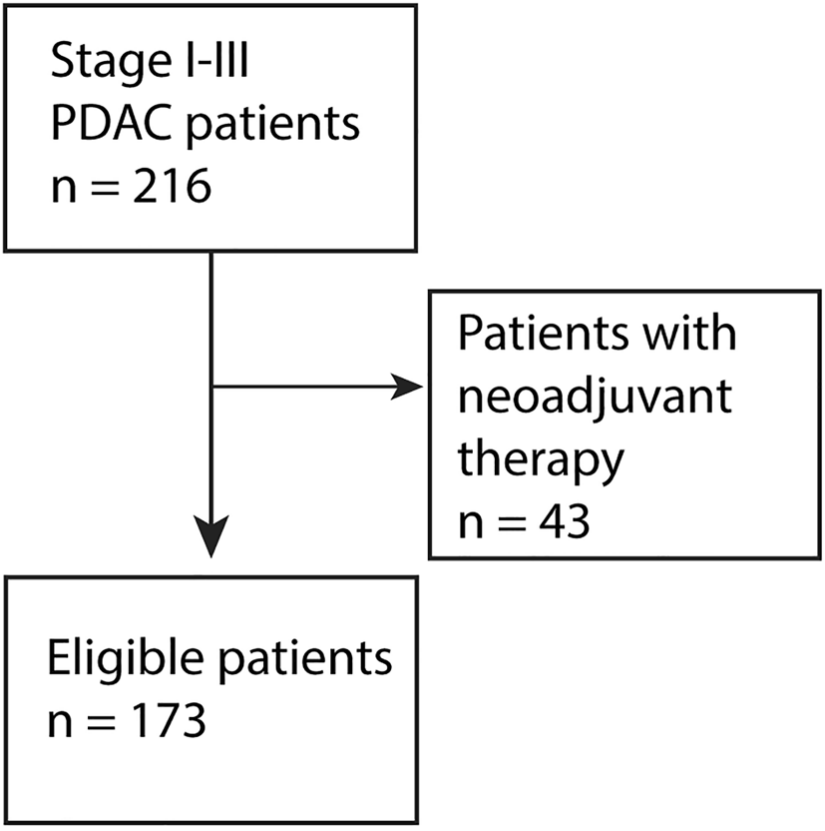
Flowchart of patient selection. We had a total of 216 patients, of which we excluded 43 patients for having received neoadjuvant therapy, leaving us with 173 study patients

Our series comprised 173 consecutive PDAC patients surgically treated at the Department of Surgery, Helsinki University Hospital between 2000 and 2013. Patients with stage IV disease or earlier neoadjuvant therapy were excluded (Fig. 1). Patients with jaundice were endoscopically treated and were non-jaundiced at surgery. The patients presented with no clinical infections or inflammation when samples were gathered.

### Cytokine measurements

Preoperative serum samples were stored at − 80 °C and thawed for the first time for the purpose of this study. We analyzed 48 different cytokines by use of the Bio-Plex Pro™ Human Cytokine 27-plex Assay (#M500KCAF0Y) and the 21-plex Assay (#MF0005KMII) (Bio-Rad, Hercules, CA, USA). We had earlier determined the levels of high-sensitivity CRP (hs-CRP) in plasma and CA19-9 in serum [6].

### Statistics

We used the Cox regression proportional hazard model to assess the effect of serum values on survival and to determine which variables would best serve for evaluation of patient prognosis. The Benjamini–Hochberg procedure served in calculating adjusted *p*-values in the univariate analysis (false-discovery rate (FDR) set to 5%, p.adjust function in R). In the multivariate analyses, we also included patient characteristics: age, gender, stage, and adjuvant therapy status. We then fitted lasso (least absolute shrinkage operator) paths for the Cox regression method with tenfold cross-validation to create a penalized Cox regression model and to select potentially important cytokines for the prognostic study model (glmnet package in *R*) [10, 11]. Bootstrapping (1000 samples) allowed the selection process to determine the overall confidence level for a variable to be included in the model. This was done by calculating the proportion of bootstrapped models in which the individual variable was included in the model. Highly correlated variables (Spearman’s correlation coefficient > 0.8; IL-10, IL-12 p70, and VEGF; IL-17 and MIP-1α; IL-3 and MIG) served as linear combinations in the lasso selection process.

We built a reference prognostic model comprising age, gender, adjuvant therapy status, and serum CA19-9. Our prognostic study model included also hs-CRP and cytokine levels.

We created time-dependent ROC curves for each unpenalized model and integrated the area under the curve (iAUC) from 1 to 10 years (giving an average time-dependent AUC over the time period) to compare the two models (TimeROC package in R) [12]. Bootstrapping (1000 samples) allowed us to determine 95% confidence intervals for the iAUCs. The assumption of the linear risk of a variable was evaluated by construction of restricted cubic splines and tests of the nonlinear component with the Cox regression (rms package in R) [13]. For variables with a suspected nonlinear HR association, if suitable, one cutoff value served to create a binary variable. To demonstrate the models’ function, we used both models to divide our patients into two groups, setting the cutoff point at the maximum value of Youden’s index in the time-dependent ROC curve at 7.5 years.

## Results

Among the patients, 10 (6%) had stage IA disease, 15 (9%) stage IB, 32 (18%) stage IIA, 65 (38%) stage IIB and 51 (29%) stage III disease.

Cox univariate analysis gave us significant (*p* < 0.05) results for (logarithmic) CRP (HR 1.66; 95% CI 1.22–2.27; FDR = 0.025), CA19-9 (HR 1.31; 95% CI 1.11–1.54; FDR = 0.025), and (binary) GRO-α (HR 1.81; 95% CI 1.29–2.54; FDR = 0.025). CA19-9 was also included in the reference model (Table 1). The lasso method provided the seven potential serum variables for the prognostic study model (Table 2): CTACK, MIF, IL-1β, IL-3, GRO-α, M-CSF, and SCF. The latter three were binary variables, whereas the rest were logarithmic. Cutoff points for GRO-α, M-CSF, and SCF were (log) 2,1, 1.1, and 2. CTACK, MIF, IL-3, GRO-α, M-CSF, and SCF had hazard ratios above 1.00, and IL-1β had a hazard ratio below 1.00.

**Table 1:** Reference model variables and Cox multivariate analysis

Multivariate analysis		95% CI			
		HR	Lower	Upper	*p*-value
Age, years					
< 65	1.00				
≥ 65	1.16		0.76	1.75	0.494
Sex					
Male	1.00				
Female	1.15		0.81	1.63	0.434
Stage*	**LNR**				
IA–IIA		1.00			**0.000**
IIB, III	< 20%	1.74	1.13	2.68	**0.012**
IIB, III	≥ 20%	3.30	2.02	5.37	**0.000**
Adjuvant treatment					
No		1.00			
Yes		0.62	0.43	0.89	**0.009**
** logCA19-9**		1.25	1.04	1.50	**0.016**

*Staging according to AJCC 8th edition. Significant values in bold

**Table 2:** Multivariate model, array data selected by the lasso model

Multivariate		95% CI				
		HR	(lasso HR)	Lower	Upper	*p*-value
Age, years						
< 65	1.000					
≥ 65	1.001			0.626	1.598	0.998
Sex						
Male	1.000					
Female	1.245			0.853	1.818	0.256
Stage*	**LNR**					
IA–IIA		1.000		0.000	0.000	**0.000**
IIB, III	< 20%	1.461		0.917	2.325	0.110
IIB, III	≥ 20%	3.143	(1.7; 99.1%)	1.869	5.286	**0.000**
Adjuvant treatment						
No		1.000				
Yes		0.697	(1.0; 77.5%)	0.470	1.035	0.074
Logarithmic values						
CTACK		1.661	(1.3; 71.6%)	0.660	4.180	0.281
CA19-9		1.334	(1.2; 95.6%)	1.080	1.647	**0.007**
IL-8		1.435	(1.1; 65%)	0.568	3.625	0.445
MIF		1.743	(1.2; 77.1%)	1.001	3.036	0.050
CRP		1.468	(1.3; 91.5%)	1.025	2.103	**0.036**
IL-1β		0.161	(0.7; 69.7%)	0.048	0.538	**0.003**
Binary values						
GRO-α**^1^		1.480	(1.2; 72.2%)	0.955	2.292	0.079
M-CSF**^2^		1.470	(1.1; 56.4%)	0.992	2.178	0.055
SCF**^3^		1.324	(1.1; 65%)	0.871	2.012	0.189

*Staging according to AJCC 8th edition. **Cutoff points at AUC ^1^log(125.9), ^2^log(12.5), and ^3^log(100). Significant values in unpenalized Cox regression shown in bold. The proportion of times the variable was selected into the model in bootstrapped selection process (1000 repetitions) is shown after the lasso HR value. This demonstrates how confident the selection process is to include the variable into the model

**Fig. 2:**
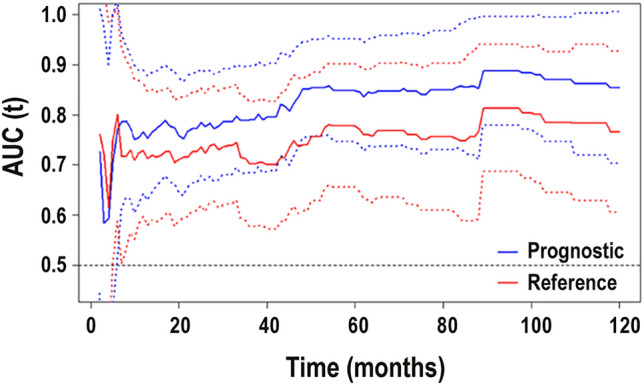
Time-dependent area under the curve for the prognostic model and reference model The integrated area under the curve (iAUC, presenting the time-averaged AUC from one to 10 years) for our prognostic model was 0.837 (95% CI 0.796–0.902) and for the reference model with the iAUC 0.759 (95% CI 0.691–0.836; no significant difference). Dashed lines represent 95% confidence intervals

For our prognostic model, the iAUC, describing the average AUC from one to ten years, was 0.837 (95% CI 0.796 –0.902), and for the reference model 0.759 (95% CI 0.691–0.836; nonsignificant difference) (Fig. 2). We also calculated how the reference model would service if combined with hs-CRP; this would result in an iAUC of 0.788 (95% CI 0.728–0.856).

**Fig. 3:**
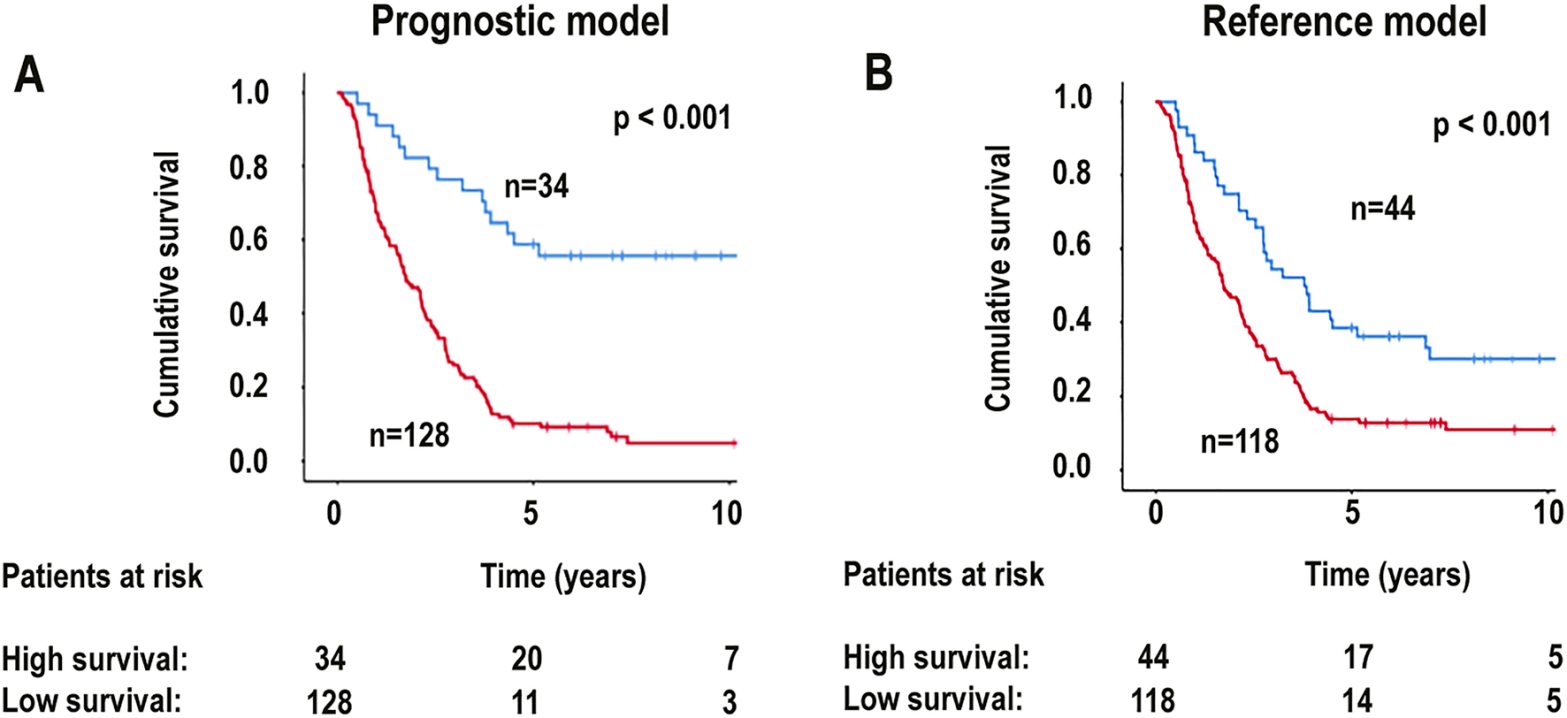
Kaplan–Meier curves Kaplan–Meier curves for high-survival (blue) and low-survival (red) patients, grouped by **A** the prognostic study model and **B** the reference model. *P*-values for the log-rank test

Both the study and the reference model served to divide the patients into two groups, based on the maximum value of Youden’s index on a time-dependent ROC curve at 7.5 years. In our prognostic study model with cytokines and CRP, the 60th percentile survival time was 4.5 (95% CI 3.7–NA) years for the predicted high-survival group and 1.3 (95% CI 1.0–1.7) years for the predicted low-survival group (log rank *p* < 0.001). The corresponding 60th percentile for survival times was 2.8 (95% CI 2.1–4.4) years (predicted high-survival group) and 1.3 (95% CI 1.0–1.7) years (predicted low-survival group) (log rank *p* < 0.001), as shown in Fig. 3.

## Discussion

We found that adding hs-CRP and inflammatory cytokines CTACK, MIF, IL-1β, IL-3, GRO-α, M-CSF, and SCF to a reference model with serum CA19-9 and patient characteristics improved the survival prediction for PDAC patients. Of these cytokines, IL-1β had a hazard ratio below 1.00, whereas the other cytokines showed hazard ratios above 1.00.

Cancer progression and poor survival are associated with pro-inflammatory cytokines [9]. In pancreatic cancer, evidence exists for changes toward a typical pro-inflammatory cytokine expression: in tissues higher MIF, IL-8, and CXCR-4 expression and reduced HLA-DR expression, and in serum higher IL-6 and IL-10 levels. Similar changes have also been evident in several other cancers. In pancreatic cancer, elevated serum IL-6 and IL-10 concentrations have been linked with worse prognosis, as has the pro-inflammatory cytokine IL-18. In tissue, higher CXCR-4 expression has been associated with metastases and reduced local expression of HLA-DR with worse survival [14].

We found serum MIF, IL-6, IL-8, IL-10, and IL-18 each to have a hazard ratio above 1.00, although only MIF and IL-8 had statistically significant results in either univariate or multivariate analysis. Another study looked at the prognostic effects of the cytokines IL-1β, IL-6, IL-8, IL-10, and TNF-α in PDAC; it showed circulating IL-8 and TNF-α to associate with poor prognosis [15]. Our results concerning the two cytokines were similar, although statistically nonsignificant.

The most widely used marker, CA19-9, has some disadvantages in clinical use. It is of both diagnostic and prognostic value [16–20]. However, those who are Lewis antigen-negative (around 5% of the population) are unable to produce CA19-9 at all [20], leading to possible false diagnoses and incorrect prognoses.

Our prognostic model incorporates pathological cancer stage, i.e., data that are available only postoperatively, but as the cytokines were measured preoperatively, our findings may prove applicable in a preoperative setting as well. With better tools to predict survival, we can treat the patients better.

All patients with jaundice were endoscopically treated before surgery. The patients presented with no infections and inflammatory states before surgery, and we excluded those with the previous neoadjuvant therapy, in order to minimize confounding bias and the risk of externally caused changes in the tumor microenvironment.

The lasso method involves sophisticated statistics incorporating variable selection, regularization, and cross-validation. It helps create the most optimal variable combination for prognostic assessment. We had no external validation set, and hence, our variable selection can be regarded primarily as an investigation to find new potential markers for survival prediction. Nonetheless, we employed bootstrapping to obtain confidence values for each selected variable and to support our findings. Our analysis involved a relatively large number of patients, 173; to our knowledge, no similar studies of this depth have yet appeared.

We hope to guide further research toward the cytokines presented here. It is not entirely understood what exactly causes these cytokines to rise. Presumably it reflects the systemic inflammatory response to the cancer, but it is possible that the elevation is caused by specific processes connected to carcinogenesis or metastasis. Further research is needed to shed light onto this. Ideally, this could lead to new targets for therapeutic cancer treatments. Furthermore, the more accurately a patient’s prognosis can be evaluated preoperatively, the better we know how to choose for them the best available treatment. Our findings may aid in the assessment of PDAC prognosis both pre- and postoperatively, and we call for further studies for validation.

## Data Availability

The authors confirm that all data supporting the findings of this study are available within the article and in its supplementary material.

## Abbreviations

hs-CRP: High-sensitivity CRP
iAUC: Integrated time-averaged area under the curve
lasso: Least absolute shrinkage operator
PDAC: Pancreatic ductal adenocarcinoma
